# Ethylene, an early marker of systemic inflammation in humans

**DOI:** 10.1038/s41598-017-05930-9

**Published:** 2017-07-31

**Authors:** Laurent M. Paardekooper, Geert van den Bogaart, Matthijs Kox, Ilse Dingjan, Anne H. Neerincx, Maura B. Bendix, Martin ter Beest, Frans J. M. Harren, Terence Risby, Peter Pickkers, Nandor Marczin, Simona M. Cristescu

**Affiliations:** 1 Department of Tumor Immunology, Radboud Institute for Molecular Life Sciences, Radboud University Medical Center, Nijmegen, The Netherlands; 2 Intensive Care Medicine, Nijmegen Institute for Infection, Inflammation and Immunity, Radboud University Medical Center, Nijmegen, The Netherlands; 3 Radboud Center for Infectious Diseases, Radboud University Medical Center, Nijmegen, The Netherlands; 4 Department of Molecular and Laser Physics, Institute of Molecules and Materials, Radboud University, Nijmegen, The Netherlands; 5 Department of Environmental Health Sciences, Bloomberg School of Public Health, The Johns Hopkins University, Baltimore, Maryland USA; 6 Department of Anaesthesia, Royal Brompton and Harefield NHS Foundation Trust, Harefield, UK; 7 Section of Anaesthesia, Pain Medicine and Intensive Care, Department of Surgery and Cancer, Faculty of Medicine, Imperial College London, London, UK

## Abstract

Ethylene is a major plant hormone mediating developmental processes and stress responses to stimuli such as infection. We show here that ethylene is also produced during systemic inflammation in humans and is released in exhaled breath. Traces of ethylene were detected by laser spectroscopy both *in vitro* in isolated blood leukocytes exposed to bacterial lipopolysaccharide (LPS) as well as *in vivo* following LPS administration in healthy volunteers. Exposure to LPS triggers formation of ethylene as a product of lipid peroxidation induced by the respiratory burst. In humans, ethylene was detected prior to the increase of blood levels of inflammatory cytokines and stress-related hormones. Our results highlight that ethylene release is an early and integral component of *in vivo* lipid peroxidation with important clinical implications as a breath biomarker of bacterial infection.

## Introduction

Ethylene (C_2_H_4_) is a major gaseous hormone in plants^1, 2^, mediating growth and development, as well as stress responses^3^. Its production is upregulated in response to diverse biotic (e.g. pathogen attack) and physical stressors (e.g. wounding, dehydration, chilling, ozone)^4^. Transcriptomics revealed that ethylene synthesis and signaling have been conserved for at least 450 million years, predating even the colonization of land^5^. The molecular pathway of ethylene biosynthesis in higher plants is well-established^6^; ethylene is enzymatically synthesized from methionine via the Yang cycle^4, 7, 8^.

No homologs of the plant ethylene biosynthetic enzymes are present in animals. However, there is evidence that mammals can form ethylene upon oxidative damage. In rat liver microsomes, copper treatment resulted in ethylene release^9^. This effect could be blocked by the lipophilic radical scavenger vitamin E, suggesting that ethylene is a product of lipid peroxidation^9^. Indeed, the oxidation of unsaturated fatty acids in suspension can lead to acyl chain decomposition and formation of small alkenes including ethylene^10, 11^. Exposing rats to nickel^12^ or mice to carbon tetrachloride or δ-aminolevulinic acid^13^ led to detectable ethylene levels in their breath. The human relevance of these model experiments is now emerging as well. We previously showed that humans can produce ethylene as a result of oxidative damage. After exposure to UV light, ethylene was found in exhaled breath and emanating from the skin of healthy volunteers^14–16^. In patients undergoing cardiac surgery, ethylene was released during specific moments of biological stress such as skin and tissue incision by diathermy or unprotected reperfusion of regional myocardial ischemic tissue^17^. Thus, ethylene can be formed *in vivo* as a product of oxidative damage.

Oxidative stress induced by reactive oxygen species (ROS) is a key component of the mammalian innate immune system during infection and (systemic) inflammation^18, 19^. One of the most fundamental mechanisms involving oxidative stress is the respiratory burst, occurring when neutrophils and monocytes encounter bacterial or fungal infections. During this burst, large amounts of ROS are rapidly produced by NADPH oxidases^20, 21^. However, the effects of this response are indiscriminative, affecting both invading pathogens and host tissues. We hypothesized that ethylene is formed as part of endogenous lipid peroxidation caused by the respiratory burst and can be detected as a gaseous signature of systemic inflammation in exhaled breath.

To test this hypothesis, we firstly utilized an isolated lipid model to consolidate basic chemical mechanisms of ethylene release by lipid peroxidation. We also investigated ethylene release and lipid peroxidation in isolated leukocytes activated by bacterial lipopolysaccharide (LPS) as a cellular model of infection. Finally, we evaluated ethylene release *in vivo* using the experimental human endotoxemia (LPS administration in healthy volunteers), a well-characterized model of systemic inflammation. We compared exhaled ethylene with established biomarkers of endogenous lipid peroxidation and aimed to clarify the temporal and mechanistic relationship between ethylene release and other biomarkers of the inflammatory stress response.

## Results

### Peroxidation of unsaturated lipids leads to ethylene formation

**Figure 1:**
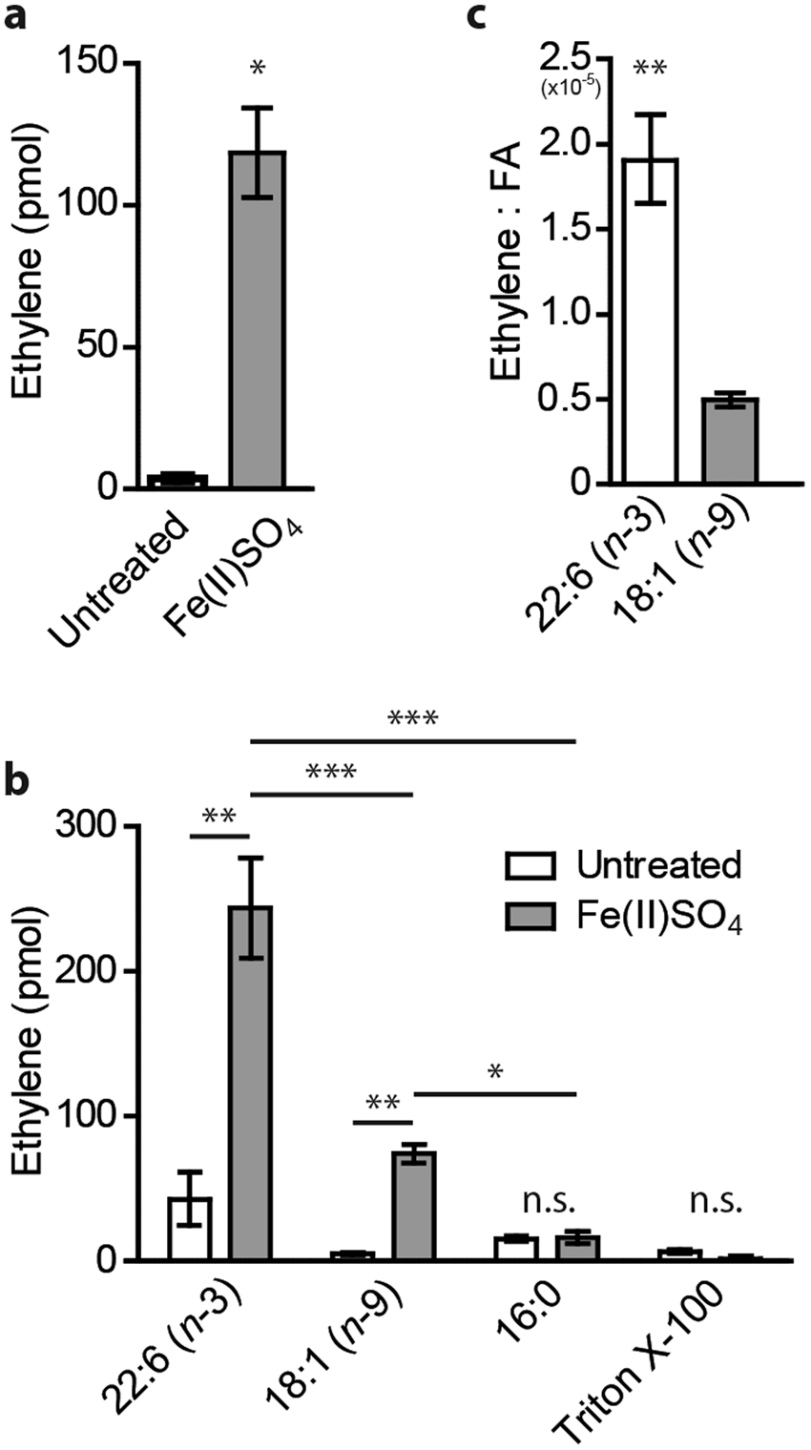
Ethylene forms upon peroxidation of unsaturated lipids. (**a**) Ethylene released from pig brain L-α-phosphatidylethanolamine (PE) suspended in milliQ H_2_O with 0.1% Triton X-100 and in the presence or absence of 500 µM Fe(II)SO_4_ after 30 minutes incubation (n = 3). **(b)** Ethylene released from *cis*-4,7,10,13,16,19-docosahexaeinoic acid (22:6 (*n*-3)), *cis*-9-Octadecenoic (18:1 (*n*-9)) or palmitic acid (16:0) fatty acids suspended in milliQ H_2_O with 0.1% Triton X-100 and 500 µM Fe(II)SO_4_ after 30 minutes incubation (n = 3). Triton X-100: detergent control with 0.1% Triton X-100 (n = 3). **(c)** Ratio of ethylene released per molecule of fatty acid (FA) for *cis*-4,7,10,13,16,19-docosahexaeinoic acid (22:6 (*n*-3)) and *cis*-9-Octadecenoic acid (18:1 (*n*-9)). Bar graphs show mean with SEM, asterisks indicate significance compared to untreated samples.

Initially, we investigated whether the *in vitro* peroxidation of mammalian lipids directly leads to formation of ethylene. We resuspended L-α-phosphatidylethanolamine (PE) extracted from pig brain in water containing the surfactant Triton X-100 and treated this suspension with Fe(II)SO_4_, a catalyst of Fenton oxidation^22^. Brain PE consists of approximately 30% polyunsaturated and 25% monounsaturated fatty acids (Avanti polar lipids). An incubation of 30 minutes resulted in significant ethylene release (p = 0.02) (Fig. 1a).

In order to explore which fatty acids caused the ethylene formation after decomposition, we performed experiments with docosahexaenoic acid (DHA, 22:6(*n*-3)), oleic acid (18:1(*n*-9)) and palmitic acid (16:0). Exposure of pure docosahexaenoic acid (DHA, 22:6(*n*-3)) and oleic acid (18:1(*n*-9)) to Fe^2+^ ions also generated significant amounts of ethylene (p < 0.01), while palmitic acid (16:0) showed no difference (p = 0.82) (Fig. 1b). Since the fatty acids were completely peroxidized after 30 minutes, we calculated the ethylene released per fatty acid, which was approximately 2 × 10^−5^ mole ethylene per mole DHA and 0.5 × 10^−5^ mole ethylene per mole oleic acid, respectively (Fig. 1c). In conclusion, peroxidation of unsaturated lipids leads to formation of ethylene.

### Lipid peroxidation, ethylene release and inflammatory cytokine production in immune cells

In order to expand to biological systems relevant to infection and inflammation, we investigated lipid peroxidation, ethylene release and inflammatory cytokine production in human cellular endotoxemia models. Monocyte-derived dendritic cells (moDCs) acquired from healthy donor blood were utilized to study basic mechanisms of lipid peroxidation and cytokine release in response to either known inducers of lipid peroxidation or LPS. MoDCs contain the NADPH oxidase NOX2, which is activated via LPS, leading to a rapid and sustained production of ROS^23–25^. Two independent assays were used to monitor lipid peroxidation. First, we used Bodipy 581/591-C11, which is a fluorescent probe mimicking unsaturated fatty acids^26^. We also investigated formation of the highly specific lipid peroxidation product 8-isoprostane with an enzyme immunoassay (EIA)^27^. In addition, we measured secretion of IL-6, a prototype cytokine associated with cellular activation. As positive controls for lipid peroxidation, we treated moDCs with Fe(II)SO_4_.

**Figure 2:**
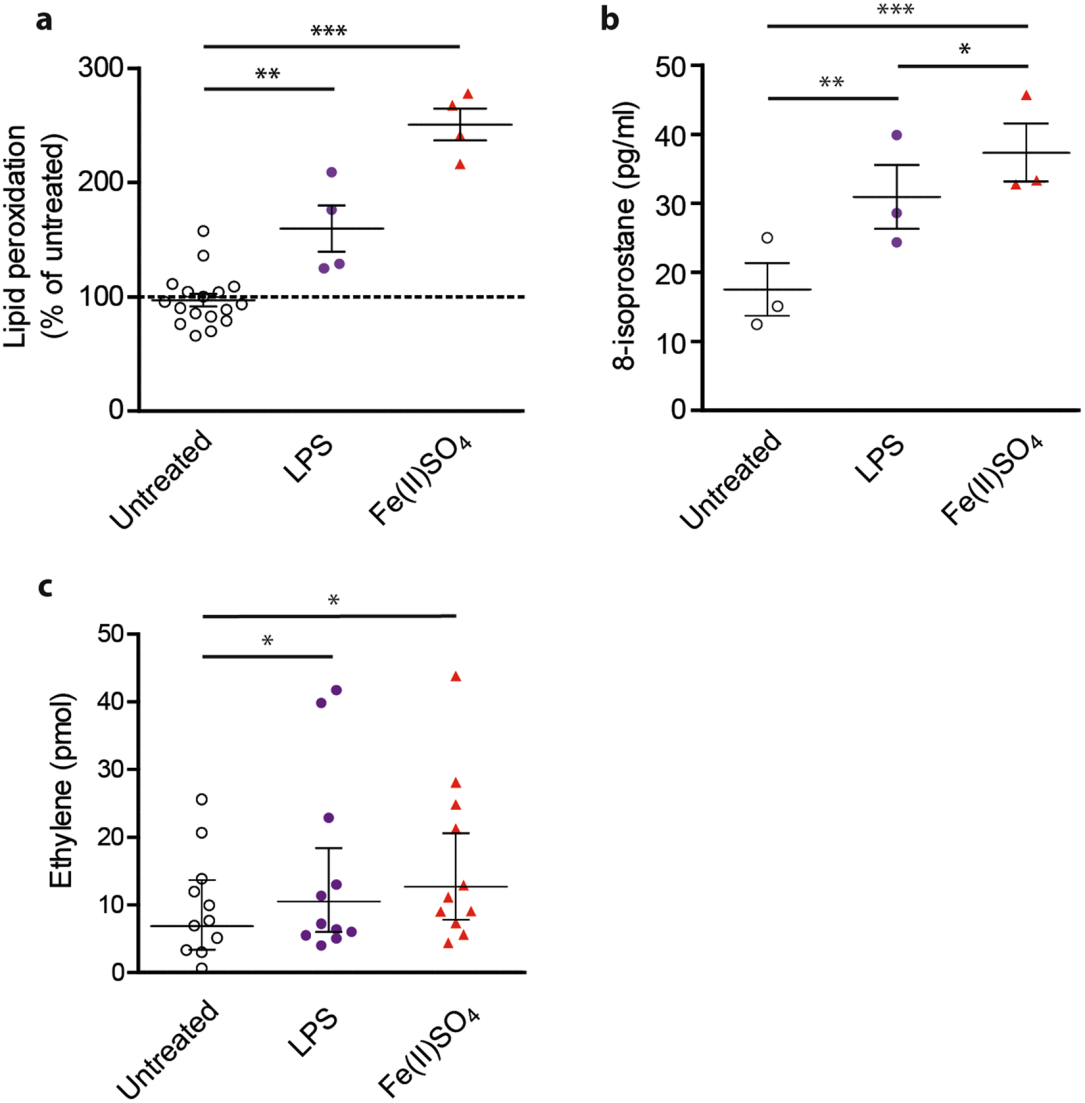
LPS triggers lipid peroxidation and ethylene formation in leukocytes. (**a**) Lipid peroxidation in moDCs as measured by blue-shift of Bodipy581/591-C11 fluorescence. MoDCs were treated with 1 µg/ml LPS or 100 µM Fe(II)SO_4_ (n = 4). **(b)** Lipid peroxidation in moDCs measured by EIA assay for 8-isoprostane. Cells were treated with either LPS or 100 µM Fe(II)SO_4_ in serum-free RPMI-1640 (n = 3). **(c)** Total ethylene released during 1 hour stimulation of leukocytes with either 1 µg/ml LPS or 100 µM Fe(II)SO_4_, as detected by laser-based photoacoustic spectroscopy (n = 11). Data points show mean with SEM (panels a and b) or geometric mean with 95% CI (panel c), asterisks indicate significance compared to untreated samples.

Exposure of moDCs to Fe(II)SO_4_ led to a significant increase in Bodipy fluorescent intensity at 510 nm (p < 0.001) (Fig. 2a). It also resulted in formation of 8-isoprostane (p < 0.001), confirming significant oxidative stress and lipid peroxidation in these cells (Fig. 2b). Treating the moDCs for 1 hour with LPS also led to a blue-shift of the Bodipy 581/591-C11 probe (p = 0.004) (Fig. 2a). This response was attenuated by the lipophilic antioxidant vitamin E (α-tocopherol) (p < 0.001, compared to LPS) and the iron chelator deferoxamine^28^ (DFO) (p = 0.005, compared to LPS), suggesting that iron content plays a role in LPS-induced lipid peroxidation (see Supplementary Fig. S1). LPS-activation of the moDCs also induced 8-isoprostane release (p = 0.004) (Fig. 2b), further demonstrating the role of lipid peroxidation in the biological effect of LPS.

The LPS treatment of moDCs resulted in significant concentration-dependent increases in IL-6 secretion with a maximum effect observed at 1 µg/ml LPS (p = 0.03) (see Supplementary Fig. S2a), while exposure to iron did not induce IL-6 production (p = 0.55) (see Supplementary Fig. S2b). Furthermore, deferoxamine or α-tocopherol did not affect IL-6 secretion either (p = 0.31; p = 0.43, respectively). Cytotoxicity data from the MTT assay indicated that, although LPS induces lipid peroxidation, this does not affect cell viability (p = 0.57), whereas Fe(II)SO_4_ does (71% viability of control, p = 0.024). Addition of α-tocopherol or deferoxamine did not affect viability (p = 0.50; p = 0.75, respectively). These results suggest that lipid peroxidation is a physiological response to LPS exposure. Our observations confirm that LPS induces both lipid peroxidation and effective cellular signaling to activate cytokine release, however lipid peroxidation alone is insufficient to activate cytokine production.

To explore the direct link between LPS, lipid peroxidation and ethylene release in human leukocytes, we stimulated peripheral blood leukocytes (PBLs) with LPS or Fe(II)SO_4_ for 1 hour and measured ethylene release by laser-based photoacoustic spectroscopy^29^ (Fig. 2c). Compared to untreated cells, both LPS and Fe(II)SO_4_ caused a significant increase in ethylene release (p = 0.04; p = 0.001, respectively). These data are supportive of the paradigm that human leukocytes produce ethylene upon LPS stimulation as a product of lipid peroxidation.

### LPS causes ethylene formation in healthy volunteers

**Figure 3:**
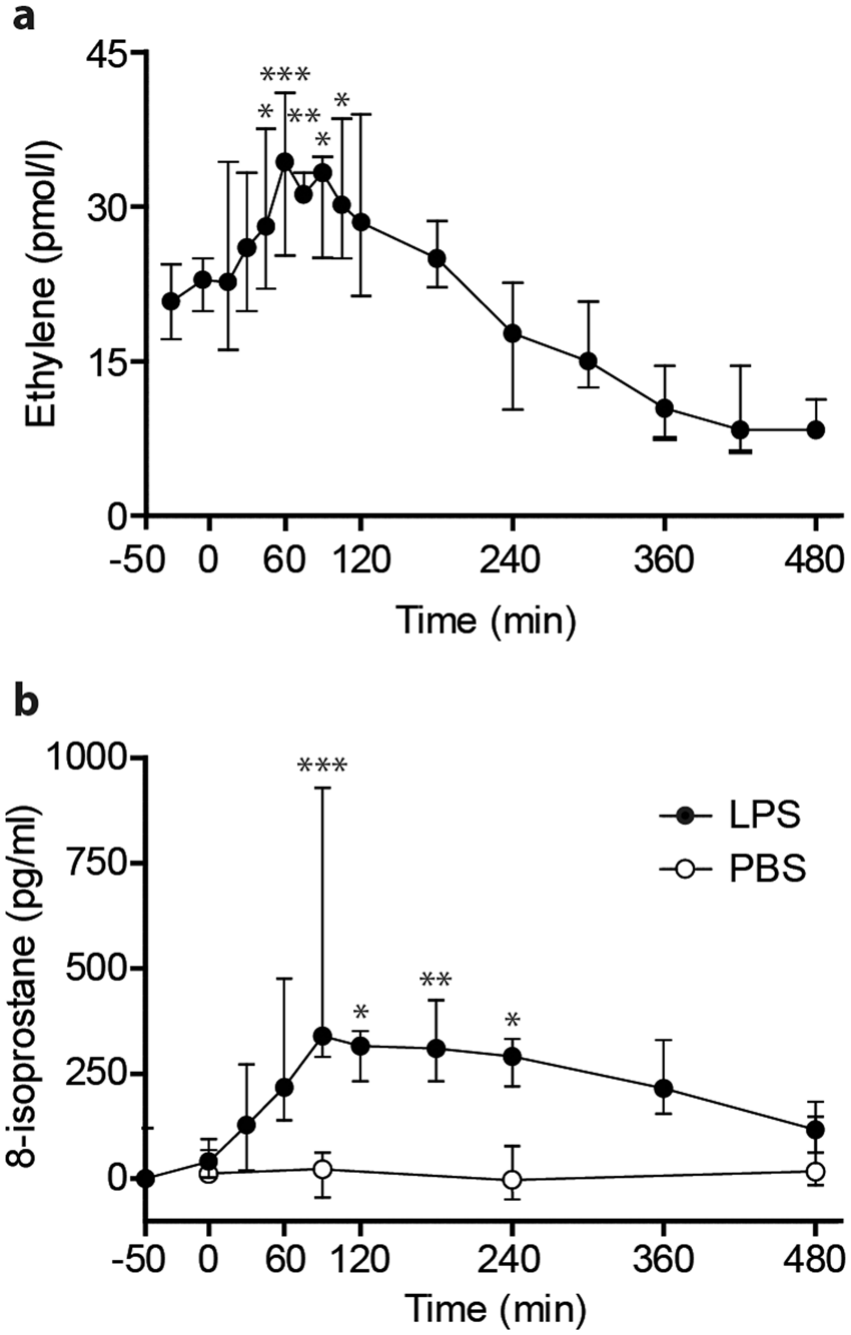
Ethylene and 8-isoprostane release during human endotoxemia. (**a**) Time course of ethylene levels in breath of healthy volunteers after intravenous administration of a single dose of LPS at t = 0. **(b)** Time course of 8-isoprostane levels as determined by EIA in serum samples of healthy volunteers after intravenous administration of a single dose of LPS (present study, n = 8) or phosphate buffered saline (PBS) (previous study, n = 6) at t = 0. Data points show median with interquartile range, asterisks indicate significance compared to baseline (t = −30 for ethylene, t = 0/−50 for 8-isoprostane PBS/LPS).

In order to determine whether systemic inflammation leads to lipid peroxidation and ethylene release *in vivo*, we utilized the experimental human endotoxemia model. This is a well characterized, controlled and reproducible human model of systemic inflammation elicited by intravenous LPS administration^30^. In all 8 subjects that underwent endotoxemia, breath ethylene levels, detected by laser-based photoacoustic spectroscopy^29^ increased rapidly after LPS administration and reached maximum levels after 60 minutes (Fig. 3a). In similar fashion, plasma levels of 8-isoprostane increased after LPS administration (Fig. 3b). Peak levels of 8-isoprostane were detected about 30 minutes after peak ethylene levels in breath. There was also a correlation between peak plasma 8-isoprostane and ethylene levels (r = 0.76, p = 0.03). To investigate the placebo effect, 8-isoprostane in plasma samples from healthy volunteers injected with phosphate buffered saline (PBS) in a previous study with identical setting as reported here, was also measured. In contrast to LPS injection, saline treatment did not result in increase of 8-isoprostane (Fig. 3b).

Interestingly, breath ethylene showed a biphasic response with levels decreasing below baseline 8 hours post-LPS, although this did not reach statistical significance. This might be caused by an increased antioxidant response, for example upregulation of glutathione levels or higher expression of ROS sequestering proteins. Another potential explanation could be auto-oxidation of NOX2. As ROS are known to react with proteins, often inactivating them in the process^25, 31^, this could self-limit the oxidative stress induced by LPS signaling.

**Figure 4:**
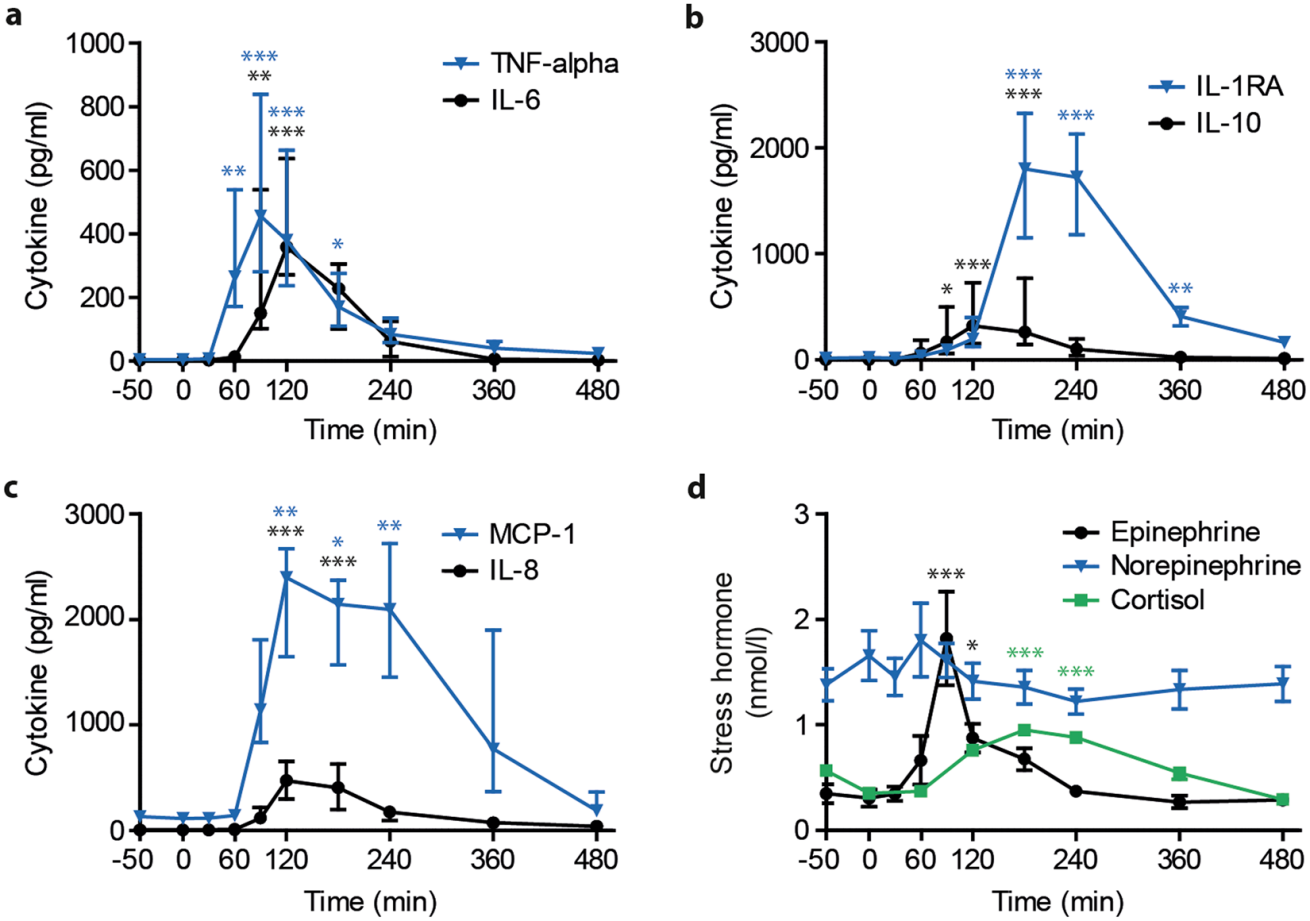
Systemic inflammation and stress response during human endotoxemia. Time course of cytokine levels as determined in plasma for **(a)** pro-inflammatory cytokines interleukin-6 (IL-6) and tumor necrosis factor-α (TNF-α), **(b)** anti-inflammatory cytokines interleukin-1 receptor antagonist (IL-1RA) and interleukin-10 (IL-10) and **(c)** chemotactic cytokines monocyte chemoattractant protein-1 (MCP-1) and interleukin-8 (IL-8) (n = 8). **(d)** Time dependent changes in plasma levels of epinephrine, norepinephrine and cortisol. Data points (n = 8) show median with interquartile range, asterisks indicate significance compared to baseline (t = −50).

Plasma levels of the pro-inflammatory cytokines/chemokines interleukin (IL)-6, IL-8, tumor necrosis factor α (TNF-α), and monocyte chemoattractant protein 1 (MCP-1) as well as the anti-inflammatory cytokines IL-10 and IL-1 receptor antagonist (IL-1RA) started to increase approximately 60 minutes after LPS administration and reached peak levels after 90–180 minutes (Fig. 4a–c). All these cytokines are well known to increase upon infection^32–35^. However, the peak levels for the inflammatory cytokines were detected at least 30 minutes later than the ethylene peak. Ethylene levels at various time-points (45 minutes, 90 minutes, and 120 minutes after LPS administration) correlated with peak levels of the archetypal anti-inflammatory cytokine IL-10 (r = 0.93, p = 0.002; r = 0.75, p = 0.03; r = 0.77, p = 0.03). No correlations between ethylene and release of IL-6 or other cytokines were found.

In order to explore ethylene release in the context of broader stress responses, we also determined plasma levels of the stress hormones epinephrine, norepinephrine and cortisol (Fig. 4d). There were no changes in norepinephrine levels over time, but epinephrine and cortisol levels increased significantly compared to their baseline, reaching peak levels at 90 and 180 minutes after LPS administration, respectively. In addition, ethylene levels at various time-points (45, 90 and 120 minutes after LPS administration) correlated with peak levels of epinephrine (r = 0.89, p = 0.007; r = 0.85, p = 0.007; r = 0.74, p = 0.04), but not with norepinephrine or cortisol.

## Discussion

Building on the well-recognized role of ethylene as a stress hormone in plants^1–4^, and previous findings linking ethylene to mammalian oxidative stress^9, 12–16^, we explored ethylene as a novel gaseous signature of the inflammatory response associated with bacterial infection. We have found that treating isolated leukocytes with LPS resulted in significant ethylene release caused by the respiratory burst. Furthermore, we show that humans produce ethylene as part of a systemic inflammatory response to bacterial endotoxin, and that this event precedes the classical inflammatory cytokine response. Collectively, these results signify ethylene as a novel biomarker for infection and oxidative stress with potentially important clinical implications.

We have conducted experiments with isolated mammalian lipids to decipher potential chemical mechanisms of ethylene formation during lipid peroxidation. Our data are relevant to both the general concept of oxidative modification of lipid membranes and to the exact process of ethylene formation during lipid peroxidation. Firstly, lipids within biological membranes are relatively vulnerable to oxidative attack compared to other cellular environments due to the high partition coefficient of oxygen in lipid membranes (≈5-fold compared to aqueous environment^36^) and due to the lack of lipophilic antioxidants (except for α/γ-tocopherol) protecting these compartments. Secondly, we propose that, during oxidative stress, ethylene production proceeds by decomposition of fatty acids by ROS. This occurs spontaneously and is not catalyzed by enzymes. In this point, this mechanism would resemble the spontaneous isomerization of 7-dehydrocholesterol to previtamin D3 in the skin induced by UV-light^37, 38^.

In order to test this novel paradigm to infection, we used leukocyte activation by LPS as a model of the inflammatory and oxidative response to infection. Leukocytes are principle mediators of host defense during all phases of infection and basic mediators of the innate immune response. They mount a strong oxidative response against invading bacteria, which is crucial for their elimination, and also as a pathological mechanism of tissue injury during uncontrolled oxidative stress^39^.

The overall aim of these cellular model experiments was to show ethylene release in an *in vitro* inflammation model and to utilize this model to forecast *in vivo* human phenomena. We have done this by characterizing lipid peroxidation and inflammatory cytokine release. Our experiments confirmed both intracellular lipid peroxidation to well-known stimuli and a clinically relevant oxidative stress biomarker response in the release of 8-isoprostane. Relevant to the inflammatory paradigm, activation of leukocytes by LPS also resulted in oxidative stress, 8-isoprostane release and ethylene production.

The human relevance of these discoveries was tested in a well characterized, controlled and reproducible model of human systemic inflammation. Remarkably, gaseous ethylene and 8-isoprostane in plasma were detected in such subjects for the first time and with important characteristics. Firstly, ethylene production started to increase rapidly following endotoxin administration to humans. It paralleled 8-isoprostane in plasma, thus providing an additional link between our *in vitro* and human data. Ethylene release appeared as part of the broader inflammatory and stress response, as we found it to correlate significantly with epinephrine and IL-10 release. The correlation with the archetypal anti-inflammatory cytokine IL-10 could indicate a potential link between endogenous lipid peroxidation and a central host defense response in triggering anti-inflammatory mechanisms. Ethylene production occurred earlier than the pro- and anti-inflammatory cytokine responses. This suggests the potential for ethylene as an early biomarker of oxidative stress in infection. For instance, the possibility of real-time breath analysis could be directly relevant to patients in intensive care units at risk of ventilator associated pneumonia^40^.

Our observation that ethylene is released prior to inflammatory cytokines also raises the exciting possibility that lipid peroxidation or ethylene itself could be involved in mounting the cytokine response. Signaling by lipid peroxidation products has been investigated before^41^ and there are some indications that ethylene itself could have a signaling function. For instance, several cell lines (of both mouse and human origin) responded with increased Ca^2+^ levels and altered gene expression after incubation with an ethylene-generating system^42^. In addition, ethylene has profound neurological effects and was widely used as an anesthetic in the 1930s, although this required very high concentrations^43^. Further studies are required to elucidate if ethylene is only a byproduct of lipid peroxidation or whether it has a signaling function.

In conclusion, we now show that humans release ethylene during systemic inflammation. The *in vitro* experiments suggested that blood leukocytes could be one of the cell types contributing to this. We could connect the chemical and biological factors causing the ethylene release in response to an inflammatory stimulus, showing that oxidation of unsaturated fatty acids plays a role in this reaction. Together, our results identify ethylene as a promising and novel biomarker for early onset of infection in the clinic.

## Methods

### Experiments with isolated lipid preparations

L-α-phosphatidylethanolamine from porcine brain (brain PE; 840022 P, Avanti), *cis*-4,7,10,13,16,19-docosahexaeinoic acid (DHA; Sigma, St. Louis, USA), *cis*-9-Octadecenoic (oleic) acid (Sigma, St. Louis, USA) and palmitic acid (Sigma, St. Louis, USA) were suspended in milliQ H_2_O containing 0.1% Triton X-100, which was deoxygenated by bubbling N_2_ through it for 15 minutes. Isolated lipid samples were placed into plastic closed-cap T25 flasks (3055, Corning, Corning, USA) that were previously equilibrated by exposure to filtered air for >48 hours at 37 °C. Brain PE, oleic acid and palmitic acid were suspended at 1.4 g/l, while DHA was suspended at 0.7 g/l. To initiate lipid peroxidation, Fe(II)SO_4_ (Sigma, St. Louis, USA) was added to a final concentration of 500 µM in 3 ml total volume. After Fe(II)SO_4_ addition, the flasks were closed to allow gas accumulation in the headspace of the samples for 30 minutes before connecting to the ethylene detector.

### *In vitro* experiments with cells

#### Cells

Human peripheral blood leukocytes (PBLs) and monocyte derived dendritic cells (moDCs) were derived from peripheral blood mononuclear cells (PBMCs) obtained from buffycoats of healthy individuals as described previously^44^ and according to institutional guidelines. Briefly, PBLs were separated from monocytes by a 1 hour adhesion step at 5% CO_2_ and 37 °C in RPMI-1640 (Thermo Fisher Scientific, Waltham, USA) with 2% human serum. Monocytes were differentiated into moDCs by culturing for 6 days at 5% CO_2_ and 37 °C in RPMI-1640 (Thermo Fisher Scientific, Waltham, USA) with 300 U/ml IL-4, 450 U/ml GM-CSF, 10% fetal bovine serum (FBS; Greiner Bio-one, Kremsmünster, Austria), 2 mM UltraGlutamine (Lonza, Basel, Switzerland) and 1% Antibiotic-Antimycotic (Gibco by Life Technologies, Kremsmünster, Austria).

#### Lipid peroxidation assays

For the Bodipy581/591-C11 assay, moDCs were activated with 2.2 mM ovalbumin, 1 μg/ml LPS and cultured in the presence of 1 μM Bodipy581/591-C11 (4,4-difluoro-5-(4-phenyl-1,3-butadienyl)-4-bora-3a,4a-diaza-s-indacene-3-undecanoic acid; Thermo Fisher Scientific, Waltham, USA) in serum-free RPMI-1640 (Gibco by Life Technologies, Kremsmünster, Austria) for 60 minutes at room temperature. 25 µM deferoxamine (Sigma, St. Louis, USA), 100 μM α-tocopherol (Sigma, St. Louis, USA) or 100 μM freshly prepared Fe(II)SO_4_ (Sigma, St. Louis, USA) was added together with the Bodipy581/591-C11. After incubation, the Bodipy581/591-C11 fluorescence was measured by FACS (excitation: 488 nm; emission: 530/30 nm; FACS Caliber, BD biosciences, Franklin Lakes, USA). The signal from unstained cells was subtracted for background correction.

For ethylene measurements, PBLs in serum-free RPMI-1640 (Thermo Fisher Scientific, Waltham, USA) were placed into plastic closed-cap T25 flasks (3055, Corning, Corning, USA) that were previously equilibrated by exposure to filtered air for >48 hours at 37 °C. Fe(II)SO_4_ (Sigma, St. Louis, USA) was added to a final concentration of 500 µM in 4 ml total volume. LPS was added to a final concentration of 1 µg/ml in 4 ml total volume. After stimulation, the flasks were closed to allow gas accumulation in the headspace of the samples for 60 minutes before connecting to the ethylene detector.

#### Cell viability assay

To assess peroxidation-induced cell death, moDCs were stimulated with 1 µg/ml LPS. 25 µM deferoxamine (Sigma, St. Louis, USA), 100 μM α-tocopherol (Sigma, St. Louis, USA) or 100 μM freshly prepared Fe(II)SO_4_ (Sigma, St. Louis, USA) was added and cells were subsequently cultured for 60 min at 37 °C and 5% CO_2_ in serum-free RPMI-1640 (Gibco by Life Technologies, Kremsmünster, Austria). The medium was aspirated and cells were incubated for 1–4 hours with 0.5 mg/ml MTT (3-(4,5-dimethylthiazol-2-yl)-2,5-diphenyltetrazolium bromide). Finally, cells were lysed in 90% (v/v) iso-propanol, 40 mM HCl and 0.0125% (w/v) SDS. Absorbance at 595 nm was read as a measure for cell viability.

#### IL-6 production assay

To assess IL-6 production, moDCs were stimulated with increasing concentrations of LPS ranging from 0.01 to 10000 ng/ml LPS, 25 µM deferoxamine (Sigma, St. Louis, USA), 100 μM α-tocopherol (Sigma, St. Louis, USA) or 100 µM Fe(II)SO_4_ (Sigma, St. Louis, USA) in serum-free RPMI-1640 (Gibco by Life Technologies, Kremsmünster, Austria) for 60 minutes at 37 °C after which supernatants were analyzed by ELISA (eBioscience, San Diego, USA) according to the manufacturer’s instructions.

### *In vivo* experiments

#### Subjects

Samples were collected from 8 healthy male volunteers (age range 19–27) participating in a randomized controlled human endotoxemia study^45^. This study was approved by the local ethics committee of the Radboud University Nijmegen Medical Centre (CMO-number 2012/455). All subjects provided written informed consent and experiments were in accordance with the Declaration of Helsinki, including current revisions, and Good Clinical Practice guidelines. All subjects were allocated to the control group, as such, they received no intervention besides the administration of lipopolysaccharide (LPS).

#### Experimental human endotoxemia

The study procedures are described in detail elsewhere^45^. Briefly, subjects were admitted to the research unit of the intensive care department where a cannula was placed in an antecubital vein to permit infusion of 0.9% NaCl solution; the subjects received 1.5 L 0.9% NaCl during one hour starting one hour before endotoxin infusion (pre-hydration) followed by 150 ml/h until 6 hours after endotoxin infusion and 75 ml/h until the end of the experiment. The radial artery was cannulated using a 20-gauge arterial catheter (Angiocath, Becton Dickinson, Sandy, Utah) and connected to an arterial pressure monitoring set (Edwards Lifesciences LLC, Irvine, CA, USA) to allow the continuous monitoring of blood pressure and blood sampling. Heart rate was recorded using a 3-lead electrocardiogram. Purified LPS (US Standard Reference Endotoxin Escherichia Coli O:113) obtained from the Pharmaceutical Development Section of the National Institutes of Health (Bethesda, MD), supplied as a lyophilized powder, was reconstituted in 5 ml saline 0.9% for injection and vortex-mixed for at least 20 minutes after reconstitution. The LPS solution was administered as an intravenous bolus injection at a dose of 2 ng/kg body weight in one minute at t = 0 hours. Subjects were continuously monitored up till 8 hours after LPS administration.

#### Breath collection

Each subject provided two single mouth-exhaled breath samples collected separately in 1-liter Mylar bags (ABC ballonnen, Zeist, The Netherlands) with 40 seconds pause in between samples. Breath samples were collected using a hand held breath sampler that is custom-built according to the ATS Guidelines and validated previously^46, 47^. The exhaled breath line consists of a bacterial filter (Air Safety Limited, Lancashire, UK), a discard bag (150 ml) for collection of the dead space, a pressure meter and ends with the sampling bag. The pressure meter provides a back pressure of 10 cm H_2_O that ensures soft palate closure and prevents nasal contamination and was calibrated for 50 ml/s exhalation flow. After a few tests on the exhalation procedure, the subjects were asked to exhale while having visual feedback from 3 LEDs (two green and one red) indicating suitable pressure/exhalation flow rate for the sampling (when both green LEDs were on). Breath samples were collected before LPS administration (approximately 5 minutes), every 15 minutes from 0 to 2 hours after LPS administration, and every hour from 4–8 hours after LPS administration. In addition, ambient air samples were collected every hour.

#### 8-isoprostane assay

8-isoprostane levels in supernatants from moDCs and in plasma samples from healthy volunteers undergoing experimental human endotoxemia were determined using an enzyme immune assay (EIA) according to the manufacturer’s instructions (Cayman Chemical, Ann Arbor, USA).

#### Cytokine/chemokine/stress hormone assays

For the determination of circulating cytokine levels from healthy volunteers undergoing experimental human endotoxemia, EDTA anticoagulated blood was centrifuged immediately after collection (2000g, 10 minutes, 4 °C), after which plasma was stored at −80 °C until analysis. Concentrations of TNF-α, IL-6, IL-8, MCP-1, IL-1RA, and IL-10 were measured using a simultaneous Luminex Assay according to the manufacturer’s instructions (Milliplex, Millipore, Billerica, MA, USA). Circulating levels of epinephrine, norepinephrine, and cortisol were determined as described previously^45^.

### Ethylene detection

Ethylene was detected by a CO_2_ laser-based photoacoustic detector (ETD-300, Sensor Sense) as described previously^29^. Briefly, the ETD-300 uses a CO_2_ laser emitting light in the 10 μm region where ethylene presents the strongest absorption features. The energy from the laser light causes local heating of the gas sample and by switching the light on and off with a certain frequency, rapid heating/cooling occurs, resulting in a periodical pressure change (a sound wave). The amplitude of this sound wave is proportional to the absorbed energy, i.e., the concentration of the ethylene in the absorption cell, and this can be detected with high-sensitivity microphones. The ETD-300 is capable of measuring ethylene on-line in the 300 pptv (pptv = parts-per-trillion volume, 1:10^12^) range within a 5 seconds time scale. All gas samples were led through a KOH-CaCl_2_ scrubber before entering the detector to remove water and carbon dioxide. The ethylene released in the headspace of the lipid samples during the 30 minute incubation was transported to the ethylene detector using clean air as carrier gas at a constant flow of 3 L/hr. Each sample was measured for 10 minutes in stop and flow mode of the instrument^48^. The PBL samples were measured in the same way, but were incubated for 60 minutes. For ethylene detection in breath of healthy volunteers, the content of the sampling bags was introduced into the ethylene detector with a small pump at a constant flow of 3 L/h. Each sample was measured for about 8 minutes (the steady state C_2_H_4_ concentration was reached within 2 min) and an averaged value of the last 2 minutes was determined. The ethylene concentration in exhaled breath was measured in duplicate for each subject and the average value used for analysis. All sampling bags were analyzed within 24 hours. The stability of exhaled ethylene in the Mylar bags was tested prior the endotoxemia experiment from 0 to 24 hours. 15 mL of ethylene from a certified calibrated mixture of 1 ppmv ethylene in air (Linde Gas Benelux, Netherlands) was injected into a Mylar bag filled with 1 L exhaled breath. Each bag was analyzed directly after injection of ethylene, and randomly after 1, 2, 4, 8, 16 or 24 hours. The experiment was performed in triplicate and showed that ethylene concentration in the bags was preserved within a 2.6% error range.

### Statistical analysis

*In vitro* results are presented as mean ± SEM or geometric mean with 95% CI of at least three different donors/independent experiments. Repeated measures one-way ANOVA with Bonferroni post-hoc tests, paired 2-sided Student’s t-tests or Wilcoxon matched-pairs signed rank tests were applied to assess significance. *In vivo* data are represented as median and interquartile range. Friedman tests with Dunn’s post-hoc test were applied to assess significance for the *in vivo* data. Furthermore, Pearson correlation on log-transformed data was used to investigate the relationship between parameters. A value of p < 0.05 was considered statistically significant (*p < 0.05, **p < 0.01, ***p < 0.001).

### Data availability

All data is available from the authors upon request.

## Electronic supplementary material

Supplementary information
