# Supplementary material for: Ethylene, an early marker of systemic inflammation in humans
Source: Sci Rep. 2017 Jul 31;7:6889. doi: 10.1038/s41598-017-05930-9 (PMC5537290; doi:10.1038/s41598-017-05930-9)
Supplement: Supplementary file 1 — Supplementary information [file 41598_2017_5930_MOESM1_ESM.pdf]

Supplementary information

Ethylene, an early marker of systemic inflammation in humans

Laurent M. Paardekooper[1], Geert van den Bogaart[1], Matthijs Kox[2,3], Ilse Dingjan[1], Anne H. Neerincx[4], Maura B. Bendix[4], Martin ter Beest[1], Frans J.M. Harren[4], Terence Risby[5,+], Peter Pickkers[2,3,+], Nandor Marczin[6,7,+], Simona M. Cristescu[4,+,*]

[1]Department of Tumor Immunology, Radboud Institute for Molecular Life Sciences, Radboud University Medical Center, Nijmegen, the Netherlands, [2]Intensive Care Medicine, Nijmegen Institute for Infection, Inflammation and Immunity, Radboud University Medical Center, Nijmegen, the Netherlands, [3]Radboud Center for Infectious Diseases, Radboud University Medical Center, Nijmegen, the Netherlands [4]Department of Molecular and Laser Physics, Institute of Molecules and Materials, Radboud University, Nijmegen, the Netherlands, [5]Department of Environmental Health Sciences, Bloomberg School of Public Health, The Johns Hopkins University, Baltimore, Maryland, USA, [6]Department of Anaesthesia, Royal Brompton and Harefield NHS Foundation Trust, Harefield, UK, [7]Section of Anaesthesia, Pain Medicine and Intensive Care, Department of Surgery and Cancer, Faculty of Medicine, Imperial College London, London, UK.

*corresponding author: Simona M. Cristescu. Email: s.cristescu@science.ru.nl

[+] senior co-authors

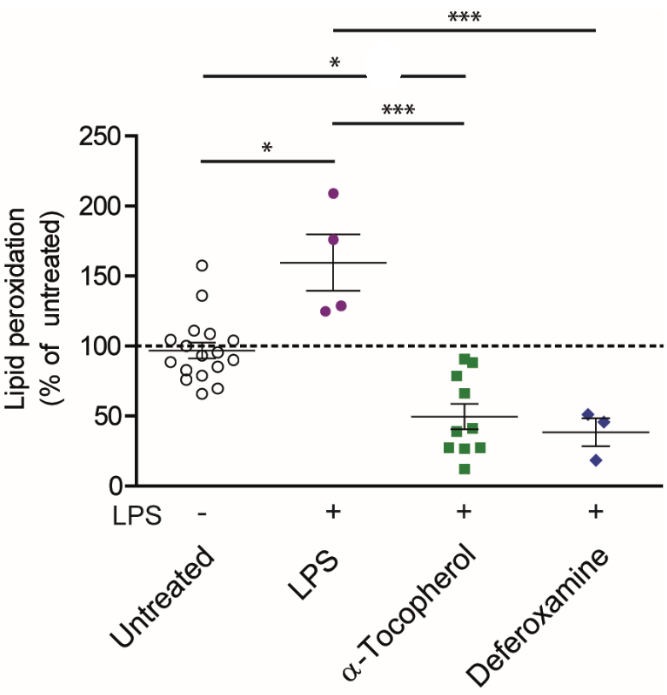

Supplementary Figure S1. Lipid peroxidation in moDCs as measured by blue-shift of Bodipy581/591-C11 fluorescence. Except for the untreated sample, all cells were exposed to 1 µg/ml LPS (data point shown in Fig. 2a). In addition, cells were treated with either 100 µM α-tocopherol or 25 µM deferoxamine. Both the lipophilic antioxidant α-tocopherol and the iron chelator deferoxamine were able to completely rescue LPS-induced lipid peroxidation ($p < 0.001$; $p = 0.005$).

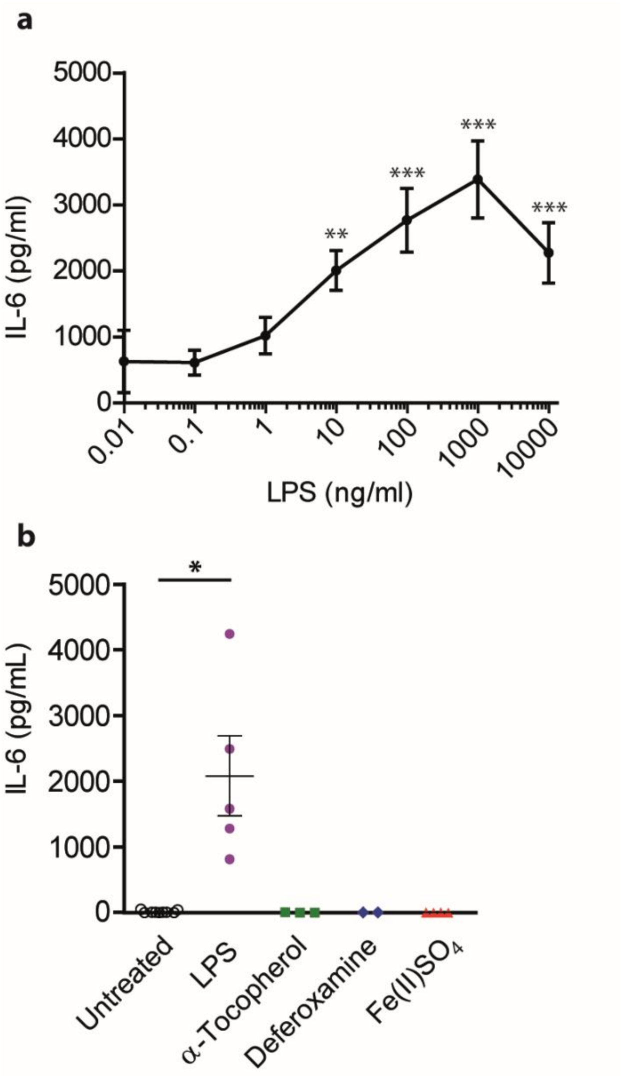

Supplementary Figure S2. (a) Production of the pro-inflammatory cytokine interleukin-6 (IL-6) from moDCs exposed to different concentrations of LPS as measured by ELISA. Error bars indicate mean +/- SEM (n=3), asterisks indicate a significant difference compared to untreated cells. **(b)** Production of IL-6 by moDCs after treatment with either 1 µg/ml LPS, 100 µM α-tocopherol, 25 µM deferoxamine or 100 µM $Fe(II)SO_4$. Only LPS was able to induce an inflammatory response ($p = 0.028$), whereas addition of either antioxidants (α-tocopherol, deferoxamine) or a chemical inducer of lipid peroxidation ($Fe(II)SO_4$) do not trigger cytokine secretion.
